# Randomized study of two different consent procedures on recall: a study within a digital alcohol intervention trial

**DOI:** 10.1186/s13063-023-07855-3

**Published:** 2024-01-02

**Authors:** Katarina Ulfsdotter Gunnarsson, Elizabeth S. Collier, Jim McCambridge, Marcus Bendtsen

**Affiliations:** 1 Department of Health, Medicine and Caring Sciences, Division of Society and Health, Linköping University, 581 83 Linköping, Sweden; 2 Perception & Design Unit, Division of Bioeconomy & Health, RISE Research Institutes of Sweden, Gothenburg, Sweden; 3 Department of Health Sciences, University of York, York, UK

## Abstract

**Introduction:**

Individuals’ comprehension of the information provided in consent forms should fundamentally influence whether to participate initially in a study and later whether to remain a participant. Existing evidence, however, suggests that participants do not thoroughly read, comprehend, or recall the information in consent forms. This study aimed to better understand how well participants recalled trial procedure information in the consent materials they received prior to taking part in a trial of a digital alcohol intervention.

**Method:**

This study was nested within an online effectiveness trial. The study included a contrast between two layout approaches to present the trial procedure information: one where all materials were shown on the same page (One page) and one where participants had to click on links to get materials for certain parts of the study information (Active request). Recall of trial procedures was measured 2 months post-randomization with four questions. Participants were also asked to leave a comment after each question.

**Result:**

Of the 2437 individuals who registered interest in the parent trial, 1197 were randomized to One page and 1240 were randomized to Active request. Approximately 90% consented to participate and 53% of the participants responded to the recall questionnaire. Contrasting the consent layout showed no marked differences between groups in three out of the four questions on recall of trial procedures. There was, however, evidence that recall of aspects of how personal data would be handled during the trial did differ between the two groups, with the Active request group reporting less recall than the One page group. Free-text comments were used to give nuance to the quantitative analysis.

**Conclusion:**

Participants exposed to different layouts of trial procedure information exhibited varying levels of information recall 2 months after consenting. The findings highlight the influence of the presentation of consent forms, which should be given attention when designing trials.

**Trial registration:**

ISRCTN ISRCTN48317451. Registered 6 December 2018, https://www.isrctn.com/ISRCTN48317451

## Introduction

Informed consent is a standard ethical component of health research when trials involve human subjects. Participants’ ability to comprehend and recall trial information should play a fundamental role when deciding whether to participate initially in a study and later whether to remain a participant. Existing evidence, however, suggests that participants do not thoroughly read, comprehend, or recall the information in consent forms [1–3]. Further, despite consent forms being a cornerstone of health and social science research for decades, the responsibility to disclose important information about a study while ensuring sufficient understanding by participants is a topic still discussed by researchers [4]. The issue of which design choices can be implemented by researchers in order to maximize consent form comprehension and information recall, and in turn increase confidence that participants’ consent truly is informed, thus remains unresolved. Such issues pose particular challenges for online research [1, 5, 6], particularly when recruitment methods are designed to be brief.

Attempts have been made to understand how participants approach the information in consent forms by, for example, examining which parts of consent forms they read most thoroughly. Evidence suggests that equal attention is not given to all components of consent forms. For instance, more participants recalled reading a target term when it was inserted into the risks or procedure section of a consent form than when the same term was present in the benefits or anonymity/confidentiality section [7]. Taken together with findings that participants tend to recall risks described in the consent form more than other information [1], it seems that individuals disproportionately attend to information concerning potential risks of studies they participate in compared to other information.

Consent form length also matters for comprehension and recall, as shorter consent forms may increase the likelihood that participants read the information [8]. Being exposed to a short consent form has also been found to increase correct responses to specific questions about a study than when exposed to a longer form [9]. However, others suggest that reducing consent material length does not necessarily lead to improved comprehension. For example, participants presented with a very short (71 words) consent form still performed poorly when asked comprehension questions about the form, and no participants opted to receive optional additional information about the study before providing consent [10]. In other words, simply shortening the form may not always have the desired effects. There are also other decisions to be made in the design of such materials. For example, one randomized study of debriefing yielded some evidence that presenting links to be clicked on was more efficacious than presenting text in an e-mail [11]. Another study found small differences in reading time according to what the study design itself was presented as [12].

Even carefully constructed consent forms often go unread. One study found that 60% of participants gave their consent to take part in the study within 30 s or less, although the consent form should have taken 2.7 min to read [13]. There is also a risk that despite a low reading and recall rate, some participants may still be under the illusion that they fully understand the information. This, in turn, may inhibit their ability to make truly informed decisions. For example, one study showed that the majority of those who reported not reading the consent forms nonetheless reported understanding the purpose and procedures of the study that they were about to participate in [14].

As data collection online becomes more common, ensuring that consent given by participants in online research can be described as informed becomes increasingly important. Although it may appear easy to obtain consent in Internet-based studies, the extent to which typical consent procedures in this context are ethically satisfactory should be examined. Indeed, evidence suggests that recall of informed consent content is similarly poor for paper-based and digital consent forms, despite participants taking slightly more time to read digitally presented forms [1]. For digital intervention studies targeting health behaviors, the challenges include conveying an accurate understanding of trial procedures, risks, and benefits. Optimization of consent forms for digital intervention studies first requires an assessment of participants’ recall of the information provided to them in this context. Therefore, this study aimed to better understand how well participants recalled trial procedure information in the consent materials they received before taking part in a trial of a digital alcohol intervention. The study included a contrast between two layout approaches of the trial procedure information.

## Method

The study was a two-arm, double-blind, parallel-group randomized trial nested within an effectiveness trial of a digital alcohol intervention [15], i.e., a study within a trial (SWAT). Both the parent study and the SWAT were prospectively registered (ISRCTN48317451), and a trial protocol including a statistical analysis plan was made available prior to trial commencement [16]. Due to the nature of this SWAT, it was not possible to ask for informed consent for participation (see for an examination of the ethical issues in these kinds of studies) [17], and thus, participants were not aware of the existence or objectives of the SWAT.

### Participants and settings

The target population was Swedish adults seeking help online to reduce their alcohol consumption. Individuals were required to be at least 18 years of age, have access to a mobile phone, and have harmful alcohol use according to Swedish guidelines. This is defined as either drinking 9 (women)/14 (men) or more standard drinks of alcohol per week (total weekly consumption) or drinking 4 (women)/5 (men) or more standard drinks on a single occasion at least once a month (heavy episodic drinking). A standard drink is in Sweden defined as 12 g of alcohol. All study materials were in Swedish, which meant that individuals who did not comprehend Swedish well enough to understand these were implicitly excluded.

Participants were recruited to the trial using web search engine advertisements (Google, Yahoo, Bing) and Facebook. Individuals interested in the study sent a text message to a dedicated phone number. Within 10 min, a response was sent back with a hyperlink to a web page which presented the informed consent material. At this stage, to address the objectives of this study, participants were randomized into one of two contrasting layouts of the consent materials. The description of materials and the two layouts are described in the following section “Interventions.” Those who consented were asked to complete a baseline questionnaire, which also assessed eligibility, and were subsequently randomized in the parent trial. Participants either received immediate access to a novel digital alcohol support tool (intervention group) or immediate access to alcohol and health information with delayed access to the novel intervention (control group).

### Interventions

Participants were randomized to one of two different layouts of presenting the informed consent information (One page or Active request). In both cases, the materials first introduced the objectives of the parent trial, which included information about being randomized to immediate and delayed access to the digital alcohol intervention. Participants receiving One page were given information on the same page on how personal information was going to be handled during and after the project, followed by information about how the data were to be analyzed and findings reported. Participants receiving Active request, on the other hand, were not shown this information on the same page but were instead presented with two links which they were told that they should click on to read more about how personal information would be handled, how their data would be analyzed, and how the findings would be reported. Thus, the only difference between groups was that Active request were asked to actively request additional information rather than having all information presented immediately. Having all consent materials on one page is common in online studies, and so the rationale for the contrast was to see if there were differences in recall when altering this standard and asking participants to request information actively, thus requiring them to interact with the materials rather than having them passively presented. Note that we did not hypothesize any direction of effect in favor of either presentation mode. Please see the Appendix for full details of the informed consent materials.

### Outcomes

Recall of trial procedure was measured 2 months post-randomization with four questions. Participants were also asked to leave a comment after each question. The information and questions presented to participants were:

### Before you accepted to join this trial you were given information about the trial procedure. We would like to ask you a few questions about this information

1. Which one of these statements most accurately describes your recall of group allocation^1^:

- I recall reading information about two groups, and that each group was going to be given access to either information or immediate access to a new mobile phone support tool.
- I recall reading information about two groups, but no details.
- I do not recall reading about allocation to two groups.

2. Which one of the groups were you allocated to?

- I was given immediate access to information.
- I was given immediate access to a new mobile phone support tool.
- I do not know.

3. With respect to how personal data would be handled during the trial, which of these do you recall reading about? You can select multiple options, and if you cannot recall any then select “I do not recall reading about personal data”:

I recall reading about:

- How data would be stored in connection to my phone number.
- How my phone number would be encrypted when stored.
- My rights to the data according to GDPR.
- Who to contact in case I have a complaint regarding data handling.
- How phone numbers were going to be treated once the project is complete.
- That my data cannot be traced to my phone number after the project is complete.
- I do not recall reading about personal data.

4. Which one of these statements most accurately describes your recall of how data collected from this trial would be analyzed and the results be made available?

- I recall reading about data analysis and communication of results, but no details.
- I recall reading about data analysis and communication of results, and some details of the analysis part.
- I recall reading about data analysis and communication of results, and some details of the communication part.
- I recall reading about data analysis and communication of results, and some details of both parts.
- I do not recall reading about data analysis nor communication of results.

### Sample size

Since the current study was a SWAT, there was no power calculation made to decide on the sample size required to detect a pre-specified effect size.

### Randomization and blinding

We used simple randomization which was fully computerized. No blocks or strata were employed. Neither participants nor research personnel were able to discover or in any way manipulate the randomization sequence. Participants and research personnel were blind to the allocation between the two informed consent layouts.

### Analysis

All analyses were done including all randomized participants, keeping them in the groups to which they were allocated (intention-to-treat). Differences among participants in One page and Active request on responses to the recall questionnaire were investigated using chi-square tests for comparison of proportions.

The free-text comments were evaluated in four steps. First, all free-text comments were read by the first and third authors (KUG and MB). Second, KUG and MB independently chose a variety of the most essential free-text comments for each question. Finally, both authors discussed all chosen free-text comments and selected the comments that captured the main content, with respect to the study objective, and illustrated response patterns.

## Result

**Fig. 1:**
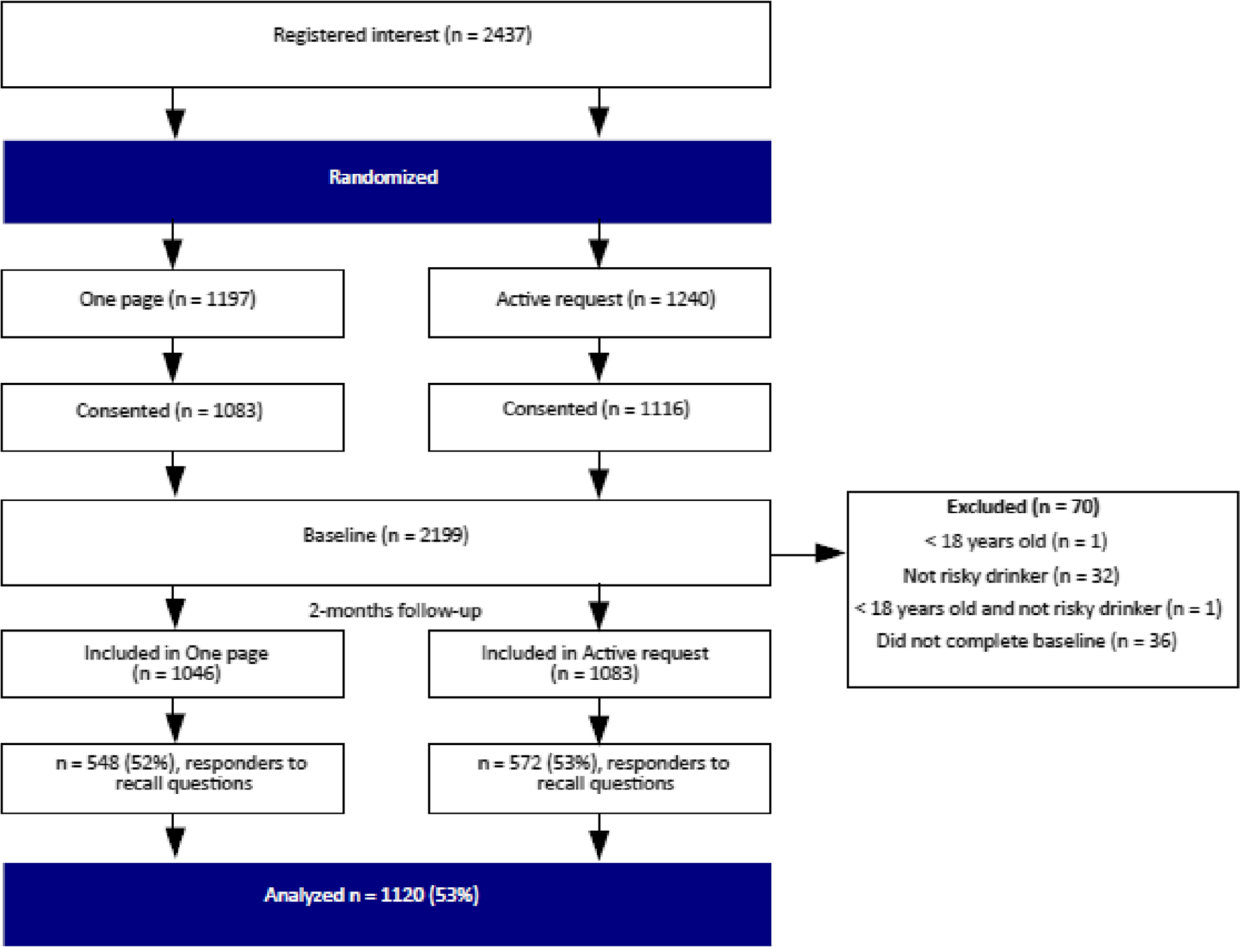
CONSORT flow diagram

Of the 2437 individuals who registered interest in the parent trial, 1197 were randomized to One page and 1240 were randomized to Active request. Consent rates were similar in both groups (90%), with a total of 2199 consenting participants. There were 70 individuals excluded from the main trial due to either not completing the baseline questionnaire or not fulfilling the trial’s inclusion criteria, leaving 1046 participants in the One page group and 1083 in the Active request group. A CONSORT flow diagram is presented in Fig. 1.

At the 2-month follow-up, the first recall question was answered by 1120 (53%) participants, and 28 free-text comments were given; the second question by 1118 (53%) with 23 free-text comments; the third question by 1122 (53%) with 24 free-text comments; and the fourth question by 1101 (52%) with 26 free-text comments. The average comment was 17 words long, ranging from a minimum of 1 word and a maximum of 127 words. Follow-up rates were comparable across the One page (*n* = 548) and Active request (*n* = 572) groups. Baseline characteristics for One page and Active request (both full groups and the subgroup of recall responders) are presented in Table 1, with little difference between the full groups and responders. Note that Table 1 presents responders to the first recall question, which includes 19 participants who did not respond to the fourth and final recall questions.

**Table 1:** Baseline characteristics of total participants and responders to the follow-up questionnaire in One page and Active request

	One page (full group) *n* = 1046	One page (responders)^a^ *n* = 548	Active request (full group) *n* = 1083	Active request (responders)^a^ *n* = 572
Total weekly consumption past week, median (IQR)	17 (10; 25)	17 (10; 24)	16 (10; 25)	15 (10; 25)
Episodes of heavy drinking past month, median (IQR)	6 (4; 12)	6 (3;10)	6 (4; 10)	6 (4; 10)
Age, median (IQR)	45 (36; 54)	48 (39; 56)	45 (35; 54)	47.5 (39; 56)
Sex, *n* (%)				
Female	629 (60%)	328 (60%)	608 (56%)	323 (56%)
Male	417 (40%)	220 (40%)	475 (44%)	249 (44%)
Household characteristics, *n* (%)				
Living with somebody with kids	363 (35%)	198 (36%)	393 (36%)	227 (40%)
Living with somebody without kids	263 (25%)	150 (27%)	281 (26%)	150 (26%)
Living alone without kids at home	221 (21%)	100 (18%)	222 (20%)	97 (17%)
Living alone with kids at home	105 (10%)	54 (10%)	110 (10%)	57 (10%)
Have a partner but not living together	94 (9%)	46 (8%)	77 (7%)	41 (7%)
Confidence^b^, median (IQR)	6 (5;8)	7 (5;8)	6 (5;8)	6 (5;8)
Importance^b^, median (IQR)	10 (9;10)	10 (9;10)	10 (9;10)	10 (9;10)
Knowledge^b^, median (IQR)	5 (3;7)	5 (3;7)	5 (2;6)	5 (3;6)
Intervention group in parent trial^c^, *n* (%)	525 (50)	248 (45)	538 (50)	282 (49)

^a^Values based on response to the first recall question. Subsequent questions saw further attrition, with 19 fewer participants in total responding to the fourth and final question

^b^Confidence, importance, and knowledge are mediators measured in the parent trial, referring to participants’ confidence in being able to make a change they specified in reducing alcohol consumption, the degree to which participants found it was important to make this change, and participants’ knowledge of how to implement actions aimed at an alcohol reduction goal

^c^Participants were randomized in the parent trial to either an intervention group (mobile phone based support) or a control group (information about alcohol and health)

### Recall of study groups and allocation

Presented in Table 2 are responses to the first and second recall question. The first question regarding participants’ recall of group allocation showed, for example, that a little more than half of participants responded that they recalled reading information about two groups and what they were going to receive, while a fifth did not recall reading about being randomized group allocation at all. The free-text comments reinforced the variation in recall and revealed some participant reactions to being allocated to the control group.

I have not received this information (control group participant)

I also remember being very disappointed not to be in the group who received support (control group participant)

**Table 2:** Frequency of responses for the first recall question: “Which one of these statements most accurately describes your recall of group allocation” and the second recall question: “Which one of the groups were you allocated to?”

Response	Total *n* (%)	One page *n* (%)	Active request *n* (%)
“Which one of these statements most accurately describes your recall of group allocation”			
I recall reading information about two groups, and that each group was going to be given access to either information or immediate access to a new mobile phone support tool.	714 (64%)	348 (64%)	366 (64%)
I recall reading information about two groups, but no details.	187 (17%)	93 (17%)	94 (16%)
I do not recall reading about allocation to two groups	219 (20%)	107 (20%)	112 (20%)
		Chi-square test: *χ*^2^(2) = 0.06, p = 0.97	
“Which one of the groups were you allocated to?”			
I was given immediate access to information	527 (47%)	266 (49%)	261 (46%)
I was given immediate access to a new mobile phone support tool	293 (26%)	134 (24%)	159 (28%)
I do not know	298 (27%)	147 (27%)	151 (26%)
		Chi-squared test: *χ*^2^(2) = 1.72, p = 0.42	

Responses to the second recall question showed that participants in both groups responded similarly. In both groups, nearly half of the participants believed they were given immediate access to information, while around a quarter believed they were given immediate access to a new mobile phone support tool.

In Table 4, responses to the second recall question and group membership in the parent trial are compared, i.e., immediate (intervention) or delayed (control) access to the digital intervention. This revealed that a greater proportion in the control group correctly identified their allocation status. Among those who did not know which group they had been randomized into, some highlighted in the free-text comments that they never received such information or had never been allocated to a group at all. The findings in Table 3 also indicate that recall of group allocation was similar in One page and Active request groups.

**Table 3:** Frequency of participants within each group of the parent trial (control and intervention) who reported believing they were allocated to the control or intervention group.

Response	Total *n* (%)	Control group *n* (%)	Intervention group *n* (%)
I was given immediate access to information	527 (47%)	377 (64%)	150 (28%)
I was given immediate access to a new mobile phone support tool	293 (26%)	29 (5%)	264 (50%)
I do not know	298 (27%)	181 (31%)	117 (22%)
		Chi-squared test: *χ*^2^(2) = 297.9, *p* < .001	
One page group			
I was given immediate access to information	266 (49%)	191 (64%)	75 (30%)
I was given immediate access to a new mobile phone support tool	134 (24%)	10 (3%)	124 (50%)
I do not know	147 (27%)	97 (33%)	50 (20%)
		Chi-squared test: *χ*^2^(2) = 159.5, *p* < .001	
Active request group			
I was given immediate access to information	261 (46%)	186 (64%)	75 (27%)
I was given immediate access to a new mobile phone support tool	159 (28%)	19 (7%)	140 (50%)
I do not know	151 (26%)	84 (29%)	67 (24%)
		Chi-squared test: *χ*^2^(2) = 141.1, *p* < .001	

Before examining responses to the third and fourth recall questions, it is worth emphasizing that since both groups were shown information about randomization and group allocation in an identical manner, we expected groups to be similar with respect to the first and second recall questions. As no statistically significant differences in responses were found for the first (*χ*^2^(2) = 0.06, *p* = 0.97) or second (*χ*^2^(2) = 1.72, *p* = 0.42) recall questions, differences in the layout of the subsequent sections of the informed consent materials did not seem to affect participants’ recall of the information presented to them earlier.

### Recall of handling of personal data

Table 4 shows responses to the third recall question. Participants could select multiple options except when selecting “I do not recall reading about personal data”, in which case the other options were deselected. The analysis revealed that, compared to Active request, more participants in One page reported recalling details about who to contact in case of a complaint and that their personal data would not be traceable to their phone numbers. Although not statistically significant, participants in One page seemed somewhat more likely to recall how their phone number was going to be treated by means of encryption during and after the study. Overall, responders allocated to Active request were more likely to report not recalling reading about personal data.

**Table 4:** Frequency of responses to the third recall question: “With respect to how personal data would be handled during the trial, which of these do you recall reading about?”

	Total *n* (%)		One page *n* (%)		Active request *n* (%)	
	Selected	Not selected	Selected	Not selected	Selected	Not selected
How data would be stored in connection to my phone number	168 (15%)	954 (85%)	84 (15%)	465 (85%)	84 (15%)	489 (85%)
			Chi-square test: *χ*^2^(1) = 0.09, *p* = 0.76			
How my phone number would be encrypted when stored	210 (19%)	912 (81%)	115 (21%)	434 (79%)	95 (17%)	478 (83%)
			Chi-square test: *χ*^2^(1) = 3.52, *p* = 0.061			
My rights to the data according to GDPR	408 (36%)	714 (64%)	193 (35%)	356 (65%)	215 (38%)	358 (62%)
			Chi-square test: *χ*^2^(1) = 0.68, *p* = 0.41			
Who to contact in case I have a complaint regarding data handling	80 (7%)	1042 (93%)	50 (9%)	499 (91%)	30 (5%)	543 (95%)
			Chi-square test: *χ*^2^(1) = 6.35, *p* = 0.012			
How phone numbers were going to be treated once the project is complete	181 (16%)	941 (84%)	101 (18%)	448 (82%)	80 (14%)	493 (86%)
			Chi-square test: *χ*^2^(1) = 4.08, *p* = 0.043			
That my data cannot be traced to my phone number after the project is complete	487 (43%)	635 (57%)	273 (50%)	276 (50%)	214 (37%)	359 (63%)
			Chi-square test: χ^2^(1) = 19.49, *p* < 0.001			
I do not recall reading about personal data	394 (35%)	728 (65%)	166 (30%)	383 (70%)	228 (40%)	345 (60%)
			Chi-square test: *χ*^2^(1) = 11.23, *p* < 0.001			

In both groups, most responders did not recall reading about specific information on how personal data would be handled. In the free-text comments, it was revealed that this was not considered an important issue by some participants. However, it was also revealed that issues regarding participants feeling safe and that their anonymity was protected were considered important.

Not a particularly important question (One page participant)

I’m not sure if I remember correctly, but I know I felt safe (One page participant)

I remember that contact would be anonymous (One page participant)

Not recalling details was also connected to being satisfied by the information presented at the time they consented by some participants:

I remember that I made an evaluation based on the information and was satisfied, so do not remember any details (One page participant)

### Recall of analysis and dissemination

Responses to the fourth recall question are presented in Table 5. No statistically significant differences in responses across groups were detected. Around one third of the responders in both One page and Active request reported that they recalled reading about data analysis and communication of results, but not any details, while slightly more did not recall reading about analysis and dissemination of results at all. In the free-text comments, some participants suggest that the information was not important, as they expected researchers to follow research ethics. It was also expressed that the anonymity was important and that no information would be reported to any government authority.

This question is of no relevance to me. I am expecting that you follow research ethics. (One page participant)

I remember that everything would be anonymous. And nothing would be reported to any government authority? (One page participant)

**Table 5:** Responses to the question: “Which one of these statements most accurately describes your recall of how data collected from this trial would be analyzed and the results be made available?”

Response	Total *n* (%)	One page *n* (%)	Active request *n* (%)
I recall reading about data analysis and communication of results, but no details	384 (35%)	189 (35%)	195 (35%)
I recall reading about data analysis and communication of results, and some details of the analysis part	67 (6%)	34 (6%)	33 (6%)
I recall reading about data analysis and communication of results, and some details of the communication part	56 (5%)	26 (5%)	30 (5%)
I recall reading about data analysis and communication of results, and some details of both parts	127 (12%)	64 (12%)	63 (11%)
I do not recall reading about data analysis nor communication of results.	467 (42%)	227 (42%)	240 (43%)
	Chi-square test: χ^2^(4) = 0.36, p = 0.99		

The comments also revealed that the focus for some participants was to join the study and obtain access to the new support, making consenting to the information without reading it an active choice.

I did not read, I just wanted to get started. (Active request participant)

My focus was on reducing my alcohol consumption, I did not focus on the study design. (Active request participant)

## Discussion

With this study, we have attempted to better understand participant’s recall of information contained in consent forms used in a randomized control trial of a digital alcohol intervention. In addition to randomization into experimental arms in the main trial, participants were also randomly assigned to receive one of two trial procedure information layouts. The only difference between the two layouts was that one layout required participants to actively request additional information by means of clicking a hyperlink rather than having all the information presented immediately.

We found that those who had to actively request more information were more likely to report not recalling reading about handling of personal data. One possible reason is that many participants in this group did not click to obtain the additional information regarding personal data, though note that a previous study found that participants were more rather than less likely to do so [11]. While we do not have any direct measurement of the number of participants who clicked the link to access the information about data handling, this result seems congruent with previous work reporting that participants did not tend to opt to obtain additional information about the study before providing consent [10].

For aspects of personal data handling where there were no statistically significant differences (“How data would be stored in connection to my phone number”, “How my phone number would be encrypted when stored“, “My rights to the data according to GDPR”), it may be that these aspects represent information that is deemed less interesting or important by participants. The findings appear consistent with previous results showing that fewer participants recalled reading a target term when it was placed within the anonymity/confidentiality or benefits section of a consent form than when it was placed in the risks or procedure section [7]. The free-text comments revealed that some participants indicate that these issues are not necessarily of high salience to them when consenting, and would appear to give consent more readily than researchers might expect or want them to do. In this study, we did see that at least some participants highlight the importance of anonymity, while in other studies participants likely neglected the details concerning participant privacy [18].

It is important to acknowledge as a study limitation that, despite the low levels of recall 2 months later, it seems reasonable that participants in this study not only read the information but also comprehended it adequately prior to making their decision to participate or not. This reliance on recall is also afflicted by social desirability considerations [19]. A related issue is that participants may have responded that they recalled specific details of the information provided simply because we asked about them, not that they recalled them. In hindsight, we recognize that we could have included a question about recall of something not in the consent materials to estimate the degree to which this happened. A strength of this study conversely, notwithstanding the potential variability in participants’ reading habits and the time they might have spent reviewing the consent forms, is the study focus on recall. Likewise, the randomized comparison of the two different layouts, which has yielded a finding that participant recall is indeed sensitive to how study information is presented.

## Conclusions

Informed consent is an important aspect of the research process as it supports the principle of individual autonomy, permitting participants control over their own decision-making about involvement in research. This study found that participants randomized to two slightly different layouts of the consent procedure showed some differences in recalling the included information. To ensure that informed consent is actually informed, future studies should continue to explore how to best design informed consent forms so that they are actually read when they are first encountered, and salient contents are remembered for as long as study participation continues.

## Data Availability

Deidentified datasets generated during and/or analyzed during the current study will be made available upon reasonable request to the corresponding author, after approval of a proposal and with a signed data access agreement.

## Appendix

**Informed consent materials**

All individuals registering interest were given information about the trial procedures prior to giving informed consent. Individuals were randomized to One page or Active request which differed in the way that the information was presented and structured. Both versions began with a general introduction and information about allocation to intervention and control, followed by information about how personal data were to be handled, and finally how data were to be analyzed and reported. However, the two latter parts of the consent information were made available through clickable hyperlinks for Active request.

**One page**

Below you will find information about the study that you should read before you consent to participate. You consent by pressing the button “I have read and give consent.” If you agree to participate in the trial, we will first ask you a series of questions.

This trial aims to estimate the effect of using a novel support tool on alcohol consumption among risky drinkers in the general population of Sweden. The tool has been developed by researchers at Linköping University. Using a mobile phone, users are asked to assess their consumption on a weekly basis and are given access to a dashboard which they can use to track their consumption, learn more about how to reduce consumption levels, and why it is important to make sure that one does not drink too much. Users will also receive motivating SMS messages throughout the week. There are no costs involved for the user. Users can at any time turn off the tool by responding to one of the SMS with the word STOP.

In order to investigate the effect of the intervention, we need to compare two groups of individuals. Therefore, all individuals who agree to participate in the study will randomly be allocated into one of two groups. One group will begin the trial by being given information that will motivate them to reduce their alcohol consumption for 4 months and then have access to the new support tool for 4 months. The other group will have these two phases reversed, thus being given immediate access to the new support tool. Regardless of which group you are allocated to, we will contact you in 1, 2, and 4 months to ask you a series of questions.

*Information about personal information*

If you decide to join the study, personal information about you will be collected and stored during the project. The purpose of the data collection is research, and public interest therefore forms a legal basis for the collection of personal information. Your responses to the baseline and follow-up questionnaires will be recorded. Your interactions with the support tool will also be stored for analysis. All your data will be connected to an encrypted version of your phone number, which means that it is necessary to know a secret key in order to identify which responses belong to which phone number. This secret key will only be known to the primary investigator of this research project (Marcus Bendtsen). When the project is complete, all phone numbers will be deleted, which means that it is no longer possible to trace an individual’s data.

Your data will be treated with confidentiality. Linköping University is responsible for your personal data. According to EU law (GDPR EU 2016/679), you have the right to access your personal data free of charge, and you are allowed to ask for changes to be made if the data is erroneous. You can also request that all your personal data be erased. Deletion of data will be done if it is deemed that it will not change the outcome of the trial. If you want to access your data, you should email the primary investigator (marcus.bendtsen@liu.se). The ombudsman can be contacted at dataskyddsombud@liu.se. If you have complaints about how your personal data has been treated, you should contact Datainspektionen.

Collection of data, and sending of SMS messages, will be handled by AlexIT AB, which is owned and run by the primary investigator (Marcus Bendtsen). Only the primary investigator (Marcus Bendtsen) will have access to the secret key used to encrypt phone numbers. Please note that once the data collection has been finalized the secret key and all phone numbers will be erased, and it will no longer be possible to trace an individual phone number to a response. All data will be removed from AlexIT AB and a final copy of the data (without phone numbers) will be stored at Linköping University. This means that the data stored is no longer considered personal data and that it is no longer possible to request access or removal of data. There is a signed agreement between Linköping University and AlexIT AB that states that AlexIT AB is allowed to help with the collection of data for research projects conducted at Linköping University (Dnr IMH-2018-00351).

In summary, after the project is completed only anonymous data will be stored. This means that it will not be possible to trace data to specific individuals, and it is therefore not possible to find your specific data on request.

*Information about how data will be analyzed and results reported*

Data collected will be analyzed at the group level; thus, no individual responses will be traceable through the analysis. To estimate the effect, and measure whether or not the effect is significant, we will create so-called regression models. These models tell us how alcohol consumption differs between those who first received the support tool and those who did not. The difference is indicative of the effect of the intervention. These models will then form a basis for a scientific publication that will contain an explanation of the trial procedure and the results that we have found.

**Active request**

Below you will find information about the study that you should read before you consent to participate. You consent by pressing the button “I have read and give consent.” If you agree to participate in the trial, we will first ask you a series of questions.

This trial aims to estimate the effect of using a novel support tool on alcohol consumption among risky drinkers in the general population of Sweden. The tool has been developed by researchers at Linköping University. Using a mobile phone, users are asked to assess their consumption on a weekly basis and are given access to a dashboard which they can use to track their consumption, learn more about how to reduce consumption levels, and why it is important to make sure that one does not drink too much. Users will also receive motivating SMS messages throughout the week. There are no costs involved for the user. Users can at any time turn off the tool by responding to one of the SMS with the word STOP.

In order to investigate the effect of the intervention, we need to compare two groups of individuals. Therefore, all individuals who agree to participate in the study will randomly be allocated into one of two groups. One group will begin the trial by being given information that will motivate them to reduce their alcohol consumption for 4 months and then have access to the new support tool for 4 months. The other group will have these two phases reversed, thus being given immediate access to the new support tool. Regardless of which group you are allocated to, we will contact you in 1, 2, and 4 months to ask you a series of questions.

For more information about how personal data will be handled, please click here: LINK (which showed the same information as for One page)

For more information about how data will be analyzed and results reported, please click here: LINK (which showed the same information as for One page).

## Footnotes

1 This refers to allocation in the parent trial. Participants were not aware of the SWAT.
